# Self-Stigma in Adults Living With Chronic Skin Disease: Development of the HautKompass Web-Based Program and Pilot Test of Its Usability, Acceptability, and Feasibility

**DOI:** 10.2196/70290

**Published:** 2025-05-22

**Authors:** Juliane Traxler, Caroline F Z Stuhlmann, Neuza da Silva Burger, Christian Stierle, Vahid Djamei, Anna Darzina, Marie Rudnik, Rachel Sommer

**Affiliations:** ^1^German Center for Health Services Research in Dermatology, Institute for Health Services Research in Dermatology and Nursing, University Medical Center Hamburg-Eppendorf, Martinistraße 52, Hamburg, 20246, Germany, 49 40 7410 54207; 2 Psychology School, Hochschule Fresenius, Hamburg, Germany; 3 Health Psychology and Paedagogy, Riga Stradiņš University, Riga, Latvia; 4 Swiss4ward, Zurich, Switzerland

**Keywords:** intervention, psychosocial care, self-stigmatization, stigma, dermatology, visible difference, eHealth

## Abstract

**Background:**

Self-stigma is common among people with chronic skin disease and can substantially impair quality of life and psychosocial well-being. Few interventions targeting skin disease–related self-stigma are available, especially in Germany.

**Objective:**

This pilot study aims to develop a web-based self-guided program to reduce self-stigma among people with chronic skin disease, and test its usability, acceptability, and feasibility.

**Methods:**

We developed the HautKompass program based on 2 systematic literature reviews and the expertise from psychodermatologists and patients. Its usability, acceptability, and feasibility were tested among adults with psoriasis, atopic dermatitis, hidradenitis suppurativa, alopecia areata, and vitiligo. After completing the program, participants provided feedback on each session and on their overall experience using the Client Satisfaction Questionnaire and study-specific feedback items. The program was considered feasible if the dropout rate was below 40% and participants spent 45 minutes or less per session. Data were analyzed descriptively.

**Results:**

HautKompass is grounded in compassion-focused therapy and cognitive behavioral therapy and consists of 8 self-guided sessions. Of the 41 persons who provided informed consent and filled in the screening questionnaire, 29 were eligible for participation. A total of 27 participants started the program and 20 completed all sessions and the posttest questionnaire. Results indicated high usability (mean 26.12, SD 6.13; on a scale ranging from 8‐32) and acceptability (mean 17.41, SD 3.12; on a scale ranging from 5‐20). Users rated the program as helpful, the psychoeducation and exercise instructions as comprehensible, and the extent of the program as adequate. Criticism concerned the length of some sessions, the electronic voice used in exercises, and some of the examples being too “general” or “cliché.” Regarding feasibility, the program’s extent was deemed adequate, participants spent substantially less time on the individual sessions (mean 16.9, SD 4.4 minutes) than the predefined criterion and few participants dropped out after starting the program (n=7, 26%), indicating low barriers. Importantly, the majority of users dropped out within the first 2 sessions, possibly due to the slightly longer duration and focus on theory, or due to unmet expectations, highlighting areas for improvement.

**Conclusions:**

Overall, HautKompass was found to be a usable, acceptable program with feasible implementation. Limitations of this pilot study include not testing accessibility for people with disabilities and the small, relatively young, and mostly female sample, which limits the generalizability of the findings. The feedback obtained was used to revise the program and the recruitment strategy prior to testing its effectiveness in a randomized controlled trial. If HautKompass is found to be effective in reducing skin disease–related self-stigma, it will be made widely available to improve psychosocial care for people with chronic skin disease and could be adapted for other skin diseases and visible differences in general.

## Introduction

People with visible differences, such as skin disease, often experience both external (or public) and internalized (or self-) stigma [1,2], which can have a range of serious consequences: low self-worth, avoidance of social situations, interference with goal achievement, and ultimately poor health outcomes and quality of life [1,3-5]. Patients, their relatives, and health care providers consider it the biggest burden in several life domains, including work and relationships [1,4,6].

To alleviate this debilitating impact, psychosocial interventions reducing self-stigma in persons with chronic skin disease are urgently needed. Systematic reviews by Topp et al [7] and Traxler et al [8] reported that most interventions including counseling, self-help, social skills training, and cognitive behavioral therapy (CBT), generally had positive effects on self-stigma and related constructs. Importantly, a range of limitations and gaps was identified in the current literature: heterogeneous study quality, insufficient description of rationale and development process, few studies on inflammatory skin diseases, and limited availability across countries and cultures. Consequently, the development of new, ideally skin-generic, psychosocial interventions and a thorough evaluation of their effectiveness are warranted.

Most interventions identified by Topp et al [7] and Traxler et al [8] require in-person contact, which has the disadvantages of potentially long waiting lists, higher costs, and greater demand from patients in terms of travel and their schedules. Many people with a dermatological condition are already burdened by frequent medical appointments and high direct and indirect costs of their disease [9-11]. Furthermore, severely affected patients in particular may not feel comfortable attending face-to-face meetings for 45 minutes or more and in the presence of a “therapist” who can be seen as a potential “stigmatizer” [12], due to their symptoms making it difficult to sit for long periods of time or due to social anxiety. Online interventions and self-guided programs can be a useful, easily accessible alternative and have the potential to reach far more individuals than face-to-face interventions [13]. Computer and web-based treatments have been studied for a variety of psychological conditions and proved effective, cost-effective, and not inferior to face-to-face therapy [13,14]. However, few online interventions are available for dermatology [15] and self-stigma, none of which are available in German.

To fill this gap, this project aimed to develop a free web-based self-guided program to reduce skin disease–related self-stigma and pilot test its usability, acceptability, and feasibility.

## Methods

### Overview

The HautKompass project, funded by the German Ministry of Education and Research (01GY2105), consists of 4 phases: (1) 2 systematic literature reviews, (2) development and pilot testing of the web-based intervention HautKompass, (3) a randomized controlled trial (RCT) to evaluate the effectiveness of the intervention (trial registration: ClinicalTrials.gov NCT06324695), and (4) implementation and dissemination. This study specifically addresses phase 2. For the pilot test, usability was defined as the extent to which HautKompass could be used as intended and with ease by patients with chronic skin disease; acceptability was defined as whether users found the HautKompass program, its content, interface, navigation, and design appealing; and feasibility was defined as whether HautKompass could be easily and conveniently implemented. Specifically, implementation was considered feasible if (1) the dropout rate from the study was below 40% [16] and (2) participants spent 45 minutes or less per session. Definitions are based on Rothstein et al [17] and the World Health Organization [18].

### HautKompass Development

**Figure 1.**
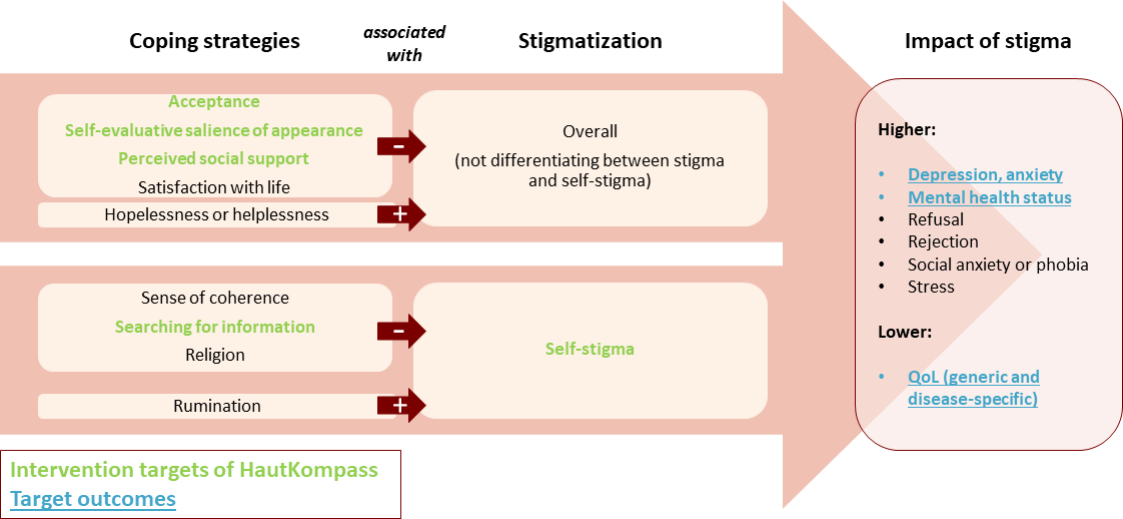
Theoretical model of determinants of stigma used in the HautKompass pilot study in German adults with chronic skin diseases. QoL: quality of life.

Initially, 2 systematic reviews were conducted to identify (1) psychosocial predictors and mechanisms of self-stigma [19], and (2) existing interventions targeting self-stigma in chronic skin disease [8]. The first review included 28 studies, which found the experience of social stigma, lack of social support, and coping strategies, such as acceptance, to be important, modifiable psychosocial determinants of self-stigma. Based on these results, a conceptual model of (self-) stigma related to chronic skin disease originally developed by Germain et al [20] was refined to be used as a therapeutic framework for HautKompass (Figure 1) [19].

To address these psychosocial determinants, HautKompass is grounded in CBT and compassion-focused therapy [21], both of which have been found to be viable and effective when delivered online [22,23]. The available literature provides the best quality evidence for CBT approaches to effectively reduce self-stigma in dermatology [24,25]. Self-compassion interventions have received little attention in the field of dermatology. However, their successful application in the field of weight-related self-stigma [26-29], their effectiveness in reducing shame and self-criticism in patients with psoriasis [30], and the association of self-compassion with adaptive coping [31] is promising for other appearance-related concerns. Furthermore, Norman et al [32] suggest that some people with visible differences, especially those who have experienced severe discrimination, might feel more comfortable with compassion-focused therapy than when purely relying on challenging unhelpful thoughts, which is the focus of CBT.

The structure and content were developed based on the 2 systematic reviews [8,19] and in consultation with a patient advisory board (n=6) as well as health care professionals with expertise in psychodermatology (n=6). Specifically, at 2 time points, patients were asked to evaluate design and content, plus to provide unstructured feedback via an online form. Health care professionals were consulted repeatedly in meetings and through email contact. According to the web-based interventions [24,33-35] identified in the systematic review [8] and expert discussion, we aimed to develop a focused program with a few, relatively short sessions so as not to exhaust the users’ attention, but with a higher frequency and flexibility than typical face-to-face therapy. Thus, the program consists of 8 sessions of approximately 15-25 minutes each, ideally completed at least once a week, which users can access from any computer and work through independently at their own pace.

**Figure 2.**
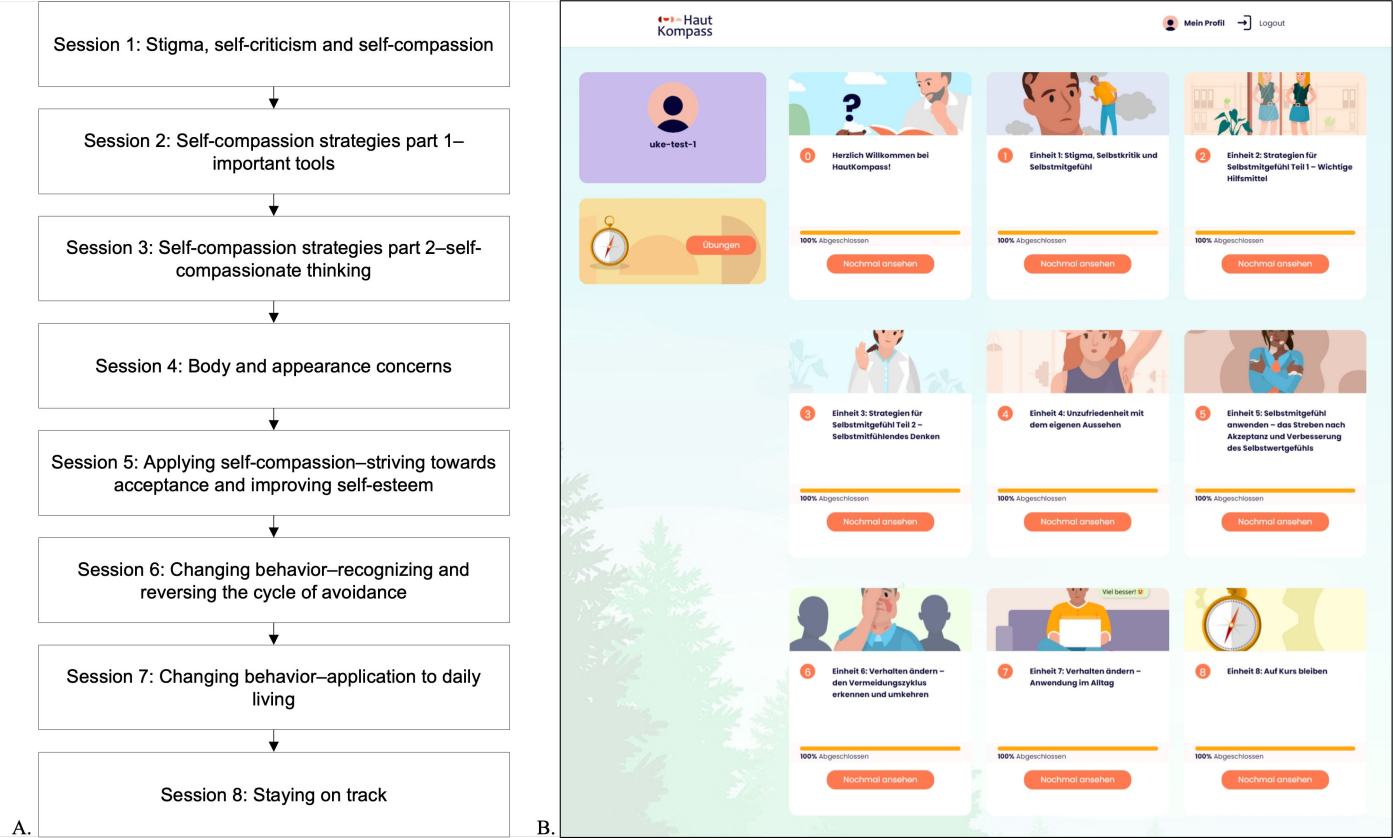
Information on the HautKompass program as used in the pilot study in German adults with chronic skin diseases. (A) Outline of the HautKompass session topics. (B) HautKompass session navigation pane. The sessions build on each other and are unlocked after completion of the previous one. Completed sessions can be reviewed at any time and selected exercises can be repeated under “Übungen” (“exercises”).

As the program was designed to be self-guided and unsupervised, it consists predominantly of psychoeducation and relatively simple exercises, including a series of breathing and imagery exercises, attention training, writing exercises, and goal setting. All content has been tailored for people with chronic skin disease. Five patient characters and 2 therapist characters have been created to guide users and to share example stories. An outline of the session topics and an illustration of the website can be found in Figure 2. Sessions and individual exercises can be repeated as needed. Users are encouraged to practice the learned techniques in their daily lives and to take breaks between sessions. Email reminders to continue with or to complete sessions were sent automatically after 2, 5, and 7 days of inactivity in the program.

### Participants and Recruitment

The target population was adults with alopecia areata, atopic dermatitis, hidradenitis suppurativa, psoriasis, or vitiligo. This selection combines indications with high disease burden and either high prevalence or particular clinical importance in dermatology. A convenience sample was recruited from the Institute for Health Services Research in Dermatology and Nursing, the dermatology outpatient clinic at University Medical Center Hamburg-Eppendorf. Adults with one of the target skin diseases were informed about the study by clinical staff and student assistants and were handed a flyer with a link and QR code to the registration page and contact details of the study team. Interested individuals could register online and receive detailed study information. This recruitment strategy may have led to selection biases, which need to be considered when interpreting the results.

Eligibility criteria were assessed through self-report using a screening form and included being 18 years or older, having one of the specified conditions, access to a stable internet and computer, proficiency in German, and the ability to consent autonomously. Participants were excluded if they received psychiatric or psychotherapeutic treatment within 12 months prior to the study, which could confound the assessment of the effectiveness of the program, or if they scored high on depression or anxiety in the screening questionnaire (see Outcome Measures section), as severely burdened individuals are recommended to seek more tailored professional help. People deemed ineligible for participation received information on how to find psychological help if needed, irrespective of the reason for which they were excluded. Enrollment closed once 5 participants per diagnosis had filled in the baseline questionnaire. Participants spending less than 5 minutes per session for more than half of the sessions were excluded post hoc from the analyses, as their feedback was deemed potentially unreliable. Participants who completed at least 1 session were considered for the analyses of the completed sessions.

### Procedure

The pilot study was conducted entirely web-based between October 2023 and December 2023. Upon enrollment, participants filled in a first questionnaire on demographics and clinical data and then received unique log-in credentials for the HautKompass website. Participants could work through the program at their own pace but were encouraged to complete it within 14 days. At the end of each session, they were asked to fill in a short postsession questionnaire concerning that specific session and could enter free text feedback. After completion of the last session, participants were asked to provide more extensive and global feedback in a posttest questionnaire.

### Outcome Measures

#### Screening Questionnaire

Patient Health Questionnaire-4 (PHQ-4) [36,37] screener consists of 4 items: 2 items that tap into depressive symptoms and 2 items for anxiety. Participants rate the degree to which they have experienced the respective complaints (eg, “feeling nervous, anxious or on edge”) over the last 2 weeks on a scale from 0=not at all to 3=nearly every day. Scores range from 0 to 12, with higher scores reflecting greater symptomatology. To screen persons before participation, a sum score of ≥5 on either the 2 anxiety items or the 2 depression items was used as a cutoff for inclusion. First, this criterion was chosen as the program was not designed for people with severe mental health problems but rather as a first low-barrier service for mildly to moderately affected individuals. Second, learning and practicing self-compassion is often difficult for individuals with clinical levels of depression and anxiety [38], especially without therapist guidance.

#### Postsession Questionnaire

Items and response scales can be found in Table 1. Usability and acceptability were each assessed by means of 4 self-developed items (eg, “The exercises in this session are useful”). Feasibility was assessed by recording dropouts and using website analytics, namely the time taken to complete each session.

**Table 1.** Postsession feedback questionnaire ratings, number of participants completing each session, and average session completion time in the HautKompass pilot study in German adults with chronic skin diseases.^a^

Item	Response scale	Values								
		S1	S2	S3	S4	S5	S6	S7	S8	all S
Usability, mean (SD)										
The session was easy to understand.	1=do not agree at all to 5=fully agree	4.70 (0.47)	4.65 (0.79)	4.59 (0.51)	4.35 (0.93)	4.76 (0.44)	4.71 (0.47)	4.63 (0.62)	4.56 (0.63)	4.58 (0.42)
The session was helpful for me personally.	1=do not agree at all to 5=fully agree	3.85 (1.09)	3.72 (1.41)	3.88 (1.36)	3.53 (1.66)	4.41 (1.00)	4.12 (1.27)	4.19 (1.17)	4.19 (1.11)	3.73 (1.10)
The exercises in this session are useful.	1=does not apply to me to 4=totally applies to me	3.20 (0.89)	3.22 (1.06)	3.12 (0.99)	2.88 (1.05)	3.65 (0.79)	3.59 (0.80)	3.50 (0.63)	3.53 (0.83)	3.19 (0.85)
The exercise instructions in this session are easy to understand.	1=does not apply to me to 4=totally applies to me	3.68 (0.58)	3.56 (0.62)	3.82 (0.39)	3.44 (0.63)	3.82 (0.39)	3.82 (0.39)	3.63 (0.50)	3.44 (0.52)	3.58 (0.45)
Acceptability, mean (SD)										
The amount of text was …	1=far too little to 5=far too much	3.20 (0.52)	3.24 (0.44)	3.41 (0.51)	3.71 (0.69)	3.00 (0.35)	3.29 (0.59)	3.44 (0.63)	3.75 (0.78)	3.37 (0.40)
The length of the session was …	1=far too short to 5=far too long	3.10 (0.45)	3.29 (0.47)	3.29 (0.47)	3.59 (0.71)	3.06 (0.24)	3.18 (0.39)	3.38 (0.5)	3.69 (0.79)	3.34 (0.38)
The session was interesting.	1=do not agree at all to 5=fully agree	4.25 (0.91)	4.17 (0.99)	4.06 (1.20)	3.71 (1.53)	4.47 (1.01)	4.18 (1.13)	4.19 (1.17)	4.38 (1.15)	4.06 (0.98)
The examples used are appropriate and helped me better understand the content of this session.	1=does not apply to me to 4=totally applies to me	3.70 (0.57)	3.17 (0.99)	3.24 (0.97)	3.00 (0.79)	3.41 (0.87)	3.59 (0.62)	3.56 (0.63)	3.50 (0.82)	3.31 (0.60)
Feasibility										
Completed, n (%)	—^b^	22 (92)	20 (83)	18 (75)	18 (75)	18 (75)	18 (75)	17 (71)	17 (71)	—
Completion time (minutes), mean (SD)	—	21.13 (25.68)	21.66 (21.70)	21.17 (19.13)	19.58 (9.39)	11.95 (5.49)	14.48 (8.75)	11.70 (4.60)	13.53 (6.90)	16.9 (4.10)

a S1–S8: Sessions 1–8; all S: averaged across all 8 sessions.

b Not applicable.

#### Posttest Questionnaire

Items and response scales can be found in Table 2. Usability was assessed by means of 5 self-developed items, 3 of which were based on those developed by Lara et al [39]. Acceptability was assessed through 2 items from Lara et al [39], 2 self-developed items, and the Client Satisfaction Questionnaire Adapted to Internet-Based Interventions (CSQ) [40]. The CSQ is an 8-item instrument (eg, “The training has met my needs”) assessing participants’ global satisfaction with internet-based interventions on a 4-point Likert scale ranging from 1=does not apply to me to 4=does totally apply to me. To compute the total score, item scores are summed and can range from 8 to 32, with higher scores indicating greater satisfaction. Feasibility was assessed through one self-developed item in the posttest questionnaire.

**Table 2.** Descriptives of posttest feedback questionnaire items (N=17)^a^ in the HautKompass pilot study in German adults with chronic skin diseases.

Item	Response scale	Values
Usability, mean (SD)		
Overall, the extent of the program was …	1=far too short to 5=far too long	3.47 (0.72)
I felt comfortable writing about my personal thoughts and experiences.	1=not true to 4=completely true	3.35 (0.70)
The website is user-friendly.^b^	1=not true to 4=completely true	3.76 (0.56)
HautKompass has met my expectations.^b^	1=not true to 4=completely true	3.35 (0.93)
HautKompass seems useful for dealing with skin disease–related self-stigma.^b^	1=not true to 4=completely true	3.53 (0.62)
Acceptability		
Is there anything you would want to change about the format of HautKompass? n (%)	Yes	5 (29)
Is there anything you would want to change about the content of HautKompass? n (%)	Yes	1 (6)
The website is persuasive, mean (SD)^b^	1=not true to 4=completely true	3.29 (0.99)
The page layout of the sessions is inviting, mean (SD)^b^	1=not true to 4=completely true	3.47 (0.72)
CSQ^c^	1=not true to 4=completely true	26.12 (6.13)
Feasibility		
Have you practiced any of the exercises and learned techniques outside the session? n (%)	Yes	14 (82)

a The posttest feedback questionnaire was completed by 20 participants, of whom 3 were excluded post hoc

b Items based on Lara et al [39].

c CSQ: Client Satisfaction Questionnaire Adapted to Internet-Based Interventions.

### Statistical Analysis

Data were processed using SPSS (version 27.0; IBM Corp). Descriptive statistics were computed for sociodemographic and clinical data as well as for the CSQ, the acceptability questionnaire, and all feedback questions.

### Ethical Considerations

The study was carried out in compliance with the Helsinki Declaration. Ethical approval was obtained from the local ethics committee at University Medical Center Hamburg-Eppendorf (approval reference: LPEK-0568). Informed consent was obtained from all participants through a written consent form, which provided details about the nature and goals of the study as well as potential harms and benefits. Participation in the study was entirely voluntary and participants retained the right to withdraw at any time without any disadvantages. Data were anonymized. Participants received a US $77.35 voucher as compensation.

## Results

### Sample Characteristics

**Figure 3.**
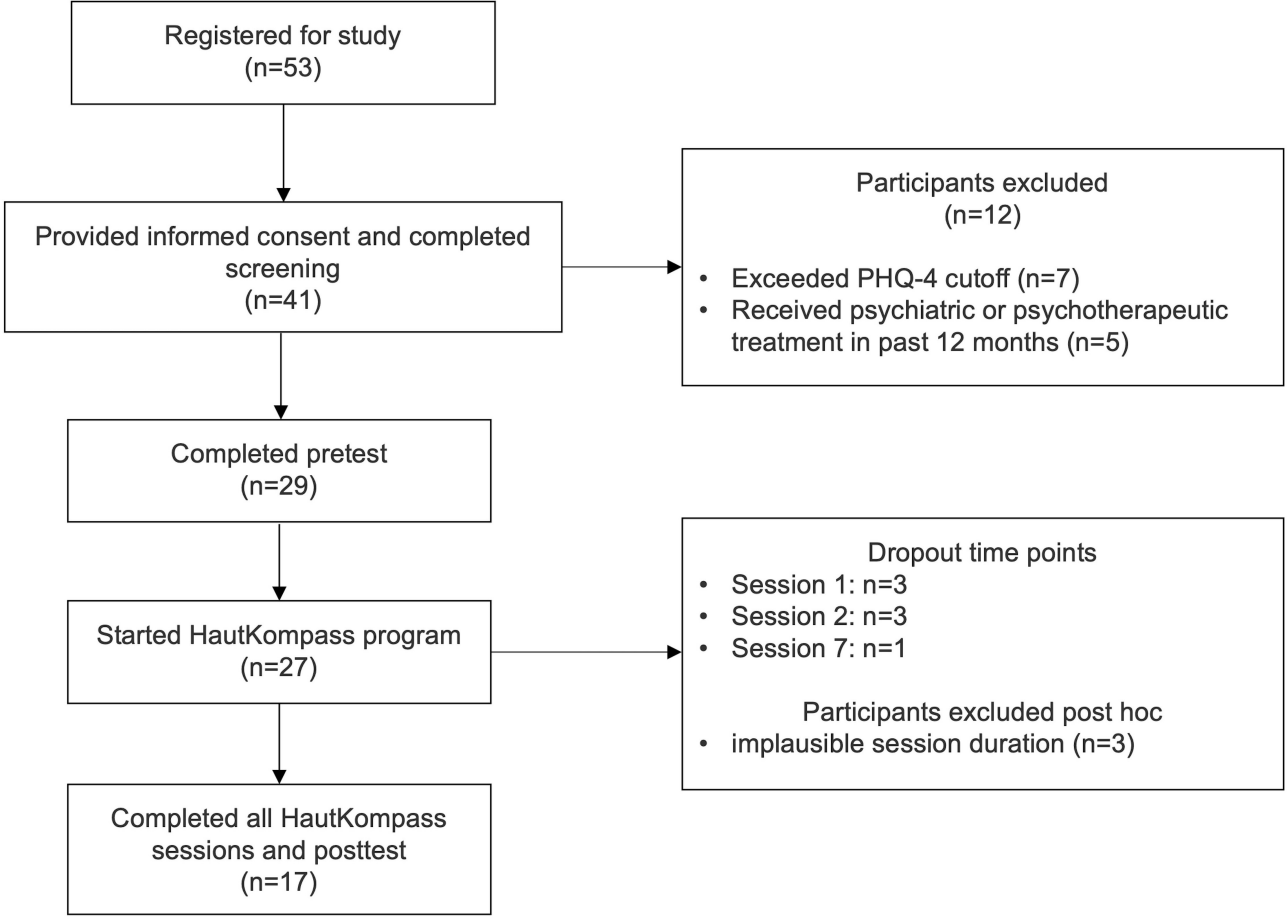
Participant flow in the HautKompass pilot study in German adults with chronic skin diseases. Three participants were excluded post-hoc from the analyses. PHQ-4: Patient Health Questionnaire-4.

Figure 3 depicts the participant flow. A total of 27 participants (n=18, 75% female) started the HautKompass program, of which 20 (74%) completed all sessions and the posttest questionnaire. Three participants completed more than half of the sessions in less than 5 minutes and, therefore, were excluded from the analysis. The demographics and clinical characteristics are presented in Table 3.

**Table 3.** Participant demographics and clinical characteristics (n=24) in the HautKompass pilot study in German adults with chronic skin diseases.

		Values
Age (years), mean (SD; range)		32.2 (12.25; 18‐63)
Sex, n (%)		
	Female	18 (75)
	Male	6 (25)
Marital status, n (%)		
	Single	7 (29)
	In relationship	11 (46)
	Married	5 (21)
	Divorced	1 (4)
Education, n (%)		
	Secondary school	9 (38)
	Vocational training	3 (12)
	Bachelor’s degree	9 (38)
	Master’s degree	3 (12)
Employment status, n (%)		
	Student	9 (38)
	Employed	14 (58)
	Unemployed	1 (4)
Skin condition, n (%)		
	Alopecia areata	1 (4)
	Atopic dermatitis	7 (29)
	Hidradenitis suppurativa	9 (38)
	Psoriasis	6 (25)
	Vitiligo	1 (4)
Current treatment, n (%)		
	Yes	21 (88)
	No	3 (12)
Duration skin condition (years), n (%)		
	Less than 1	1 (4)
	1‐5	5 (21)
	5‐10	3 (12)
	More than 10	15 (63)
Disease severity, n (%)		
	Mild	11 (46)
	Moderate	11 (46)
	Severe	2 (8)

### Usability

Tables1  2 show the participants’ responses to the postsession and posttest feedback questionnaires, respectively. The extent of HautKompass was perceived to be adequate and participants felt overall comfortable writing about their personal thoughts and experiences. Furthermore, the website was rated as very user friendly, useful for reducing skin disease–related self-stigma, and has mostly met users’ expectations. The individual sessions were overall easy to understand (mean 4.56, SD 0.42), and rather helpful for users personally (mean 3.73, SD 1.10). The exercises were perceived as mostly useful (mean 3.19, SD 0.85), and the instructions as easy to understand (mean 3.58, SD 0.45).

### Acceptability

Acceptability of HautKompass was generally high. Both layouts of the website and individual sessions were rated as likable. User satisfaction assessed with the CSQ was high, with an average score of mean 26.12 (SD 6.13, range: 9‐32). Six participants (35%) suggested making some changes to the format or content of the program, such as to “also address people who don’t think negatively of themselves all day long” and to make the examples “*less general”* or *“less cliché.”* Regarding session-specific feedback, participants rated the sessions as interesting (mean 4*.*06, SD 0.98) and considered the examples appropriate and helpful (mean 3.31, SD 0.60). The amount of text (mean 3.37, SD 0.40) and length of each session (mean 3.34, SD 0.38) were both rated as “just right” on average, although 2 sessions were rated as a bit too long and text-heavy, which also reflected in the open text feedback (Textbox 1). The open text feedback revealed some additional criticisms and suggestions but many positive features were highlighted as well.

Textbox 1. **Examples of free text feedback in the HautKompass pilot study in German adults with chronic skin diseases.**

**Criticism**

- “The feedback from the system does not address your specific case, but is very general, which is somewhat frustrating after you have put energy into the exercise.” [Session 1]
- “It would be more pleasant if a real voice were used instead of a computerized voice.” [Session 2]
- “Going through the same concept three times, even if it’s a different situation, is a bit long.” [Session 8]
- “Apart from the length, the unit was really good.” [Session 8]

**Positive comments**

- “I think the session is really very good and everything is right in terms of content. It’s easy to understand and certainly helps everyone in general. It’s also fun and motivates you to do the next session.” [Session 3]
- “It was good to take some active time and meditate / relax to calm down.” [Session 2]
- “The calendar with the planned activities is great. I have decided to have a fixed day where I do things that I like to do as a relaxation day.” [Session 3]
- “I found it very helpful to set a precise goal which I work through step by step.” [Session 7]

### Feasibility

In total, 53 persons registered for the study (female: n=34, 64%; male: n=18, 34%; diverse: n=1, 2%), of which 41 (female: n=28, 68% and male: n=13, 32%) signed the informed consent form and completed the screening questionnaire. The nonparticipation rate was higher for males (n=5, 28%) than females (n=6, 17%). At this stage, 12 persons were excluded, either due to high scores on the PHQ-4 (n=7) or due to receiving some form of psychotherapeutic treatment within the past 12 months (n=5). All of the eligible 29 persons filled in the pretest questionnaire, and 27 started working through the program. Six of them dropped out within the first 2 sessions, while only 1 participant dropped out at a later stage (dropout rate=25.93%). In total, 20 users completed all sessions and the posttest questionnaire.

On average, participants spent 16.90 minutes per session, with session 7 being the shortest (11.70 minutes) and session 2 being the longest (21.66 minutes). Furthermore, 14 participants (82.35%) practiced some of the exercises and learned techniques in their daily lives.

## Discussion

### Principal Results

The aim of this pilot study was to assess the usability, acceptability, and feasibility of the newly developed HautKompass web-based program for people experiencing skin disease–related self-stigma. Overall, all 3 criteria were found to be largely satisfactory: users found HautKompass to be acceptable and usable, and suggested a few changes, which were addressed where possible. The predefined feasibility criteria, namely a dropout rate below 40% and a duration of individual sessions below 45 minutes, were met.

Overall, HautKompass was rated as highly userfriendly and useful. In light of the relatively young sample (aged 18‐63 years), it needs to be noted that these results were obtained in a small sample of 20 individuals and may not apply to older individuals, many of whom are less adept and less confident in using online programs and might experience barriers that were not detected in this study.

The pilot users found the program largely acceptable and the overall appearance was appealing to most. A few suggestions for changes were made, which were addressed to enhance the acceptability of HautKompass: the computerized voice was replaced by real voice recordings, and some examples were slightly rephrased to be more appealing or relatable. One user indicated that receiving nonpersonalized feedback was frustrating, which aligns with previous findings that users of eHealth programs value personalized content and feedback [41,42]. Unfortunately, offering tailored feedback was not feasible within the scope of this project. In addition and as described earlier, the results may not be generalizable to male and older users. While we have deliberately designed both male and female characters of different age groups and aimed to include examples that may be relevant in different stages in life, we cannot rule out that the design and overall appearance of HautKompass appeal most to younger females. In fact, 2 users (female, 24 years and male, 33 years) raised concerns that the program seemed to be designed for a relatively young audience and that older users might not feel equally well represented. The data also suggest that it is feasible to conduct an RCT evaluating the effectiveness of HautKompass and subsequently implementing the program for broader use. It is worth noting that only half of those who registered for the study actually started the program. Twelve participants (23%) were excluded at screening, which may indicate that people with poorer mental health in particular seek this kind of help, and the exclusion criteria were too strict. Although the program was not specifically designed for individuals with severe anxiety or depression, a recent meta-analysis showed that self-compassion can alleviate depressive and anxiety symptoms also in clinical populations, even using web-based formats [43]. It was thus decided to omit the PHQ-4 criterion for the upcoming RCT. A further 12 of those who registered did not give informed consent, possibly because they were only given more detailed information about the study at this point. It is conceivable that they were either discouraged by the time commitment required for this pilot study or did not feel that the program was for them, both of which underline the need for more targeted recruitment. However, the dropout rate from the program itself (25.93%) was comparable to or even lower than in other digital interventions [16,44,45]. This shows that, once participants complete the screening, they encounter few barriers and after working through the first sessions are unlikely to dropout. The finding that the sessions during which most participants did dropout (sessions 1 and 2) were also the longest ones and may indicate the participants’ preference for short and concise sessions, which is in line with previous research [46]. Furthermore, the information provided prior to participation may have been insufficient such that some participants held expectations they realized were not going to be met when starting the program [47]. Another potential explanation is that the first session in particular was largely theoretical and offered little concrete advice for daily life. To address these issues, we shortened some sessions by omitting challenging and less useful exercises, avoiding repetitions, and reducing the amount of text, and we refined the recruitment information to better reflect the program’s content and the study procedures in order to give potential participants realistic expectations. Altogether, the short session duration, low dropout rate, and willingness of users to revise exercises still demonstrate the feasibility of implementing HautKompass as a self-guided flexible, web-based program.

### Strengths and Limitations

An important strength of the development process is its participatory approach. A patient advisory board was consulted repeatedly to tailor the program to their needs to guarantee that HautKompass would be both relevant and appealing to the target group. Furthermore, the sample covered a wide range of educational backgrounds.

This study also has several limitations that need to be addressed. The sample is rather small and not fully representative of the general population; for example, men were underrepresented and although the age range went up to 63 years, the samples were relatively young. This may indicate that men and older people do feel less addressed by the HautKompass program and the recruitment strategy. While some studies testing the effectiveness of eHealth interventions have not found substantial gender differences, men are typically less likely to seek help [39] and more likely to drop out from web-based interventions [48]. Evidence suggests older people are still more reluctant to use eHealth interventions, although the proportion of elderly users is increasing [40]. Consequently, although half of the participants in the advisory board were male and we included their perspective in the refinement of the program, the data from this pilot study did not allow us to check whether the program appeals to men and older individuals, whether they feel adequately represented and their needs are met. Hence, extra efforts to recruit these groups might be needed, for example, using stratified sampling (ie, balanced numbers of women and men, and younger and older participants) or highlighting the ease of use to less techsavvy people.

Another limitation is that we did not test accessibility for people with learning disabilities, or cognitive and motor impairments. HautKompass has many visual features, including exercises that require vision, so it may not be well-suited for people with visual impairments. While we reduced the amount of text and aimed to keep its difficulty low, wider user testing is indicated.

### Conclusions

This pilot study shows that HautKompass is a highly usable and acceptable web-based intervention, and the data suggests its implementation to be feasible. The results have highlighted several barriers and areas for improvement, which have been addressed where possible. Currently, the program’s effectiveness in reducing self-stigma is being tested in an RCT with a 6 months follow-up with 500 participants (n=100 per target diagnosis). Participants are being recruited in hospitals, clinics, and dermatology practices across Germany as well as through patient organizations. We expect HautKompass to reduce self-stigma by improving self-compassion and acceptance in people with various chronic skin diseases, compared to a waitlist control group. If proven effective, we aim to make HautKompass freely available and disseminate it nationwide through patient organizations and over 5000 practicing dermatologists. The program is the first of its kind available in the German language. As psychodermatological services are hardly available in Germany so far, this would be a major advance in psychosocial care with the potential to significantly improve patients’ quality of life and well-being.

In addition, various adaptations, extensions, and new applications of HautKompass are possible. As the program has been developed to be skin-generic, it should be tested for other skin diseases. It could also be adapted for visible differences in general with relatively few modifications. In addition, a mobile app would be of great value to give participants more flexibility in time and place, and easier access to do the exercises more frequently. Finally, the current version of HautKompass is aimed at adults, but children and adolescents are still developing their self-image and self-esteem and maybe even more vulnerable to the detrimental effects of self-stigma. Therefore, there is great potential in developing a version for younger patients, parents, or relatives of children affected by skin disease.

## Abbreviations

CBT: cognitive behavioral therapy
BMBF: Bundesministerium für Bildung und Forschung (German Federal Ministry for Education and Research)
CSQ: Client Satisfaction Questionnaire Adapted to Internet-Based Interventions
PHQ-4: Patient Health Questionnaire-4
RCT: randomized controlled trial

## References

[1] Sommer R, Augustin M, Mrowietz U, Topp J, Schäfer I, von Spreckelsen R (2019). Perception of stigmatization in people with psoriasis-qualitative analysis from the perspective of patients, relatives and healthcare professionals. Hautarzt.

[2] Alpsoy E, Polat M, FettahlıoGlu-Karaman B (2017). Internalized stigma in psoriasis: a multicenter study. J Dermatol.

[3] Corrigan PW, Rao D (2012). On the self-stigma of mental illness: stages, disclosure, and strategies for change. Can J Psychiatry.

[4] Temel AB, Bozkurt S, Alpsoy E (2017). 029 Internalized stigma in acne vulgaris, vitiligo and alopecia areata. J Investig Dermatol.

[5] Krüger C, Schallreuter KU (2015). Stigmatisation, avoidance behaviour and difficulties in coping are common among adult patients with vitiligo. Acta Derm Venereol.

[6] van Beugen S, Maas J, van Laarhoven AIM (2016). Implicit stigmatization-related biases in individuals with skin conditions and their significant others. Health Psychol.

[7] Topp J, Andrees V, Weinberger NA (2019). Strategies to reduce stigma related to visible chronic skin diseases: a systematic review. J Eur Acad Dermatol Venereol.

[8] Traxler J, Stuhlmann CFZ, Graf H, Rudnik M, Westphal L, Sommer R (2024). Interventions to reduce skin-related self-stigma: a systematic review. Acta Derm Venereol.

[9] Jungen D, Augustin M, Langenbruch A (2018). Cost-of-illness of psoriasis—results of a German cross-sectional study. J Eur Acad Dermatol Venereol.

[10] Kullab J, Schielein MC, Stuhlmann CFZ (2023). Out-of-pocket costs in alopecia areata: a cross-sectional study in German-speaking countries. Acta Derm Venereol.

[11] Mohr N, Naatz M, Zeervi L (2021). Cost-of-illness of atopic dermatitis in Germany: data from dermatology routine care. J Eur Acad Dermatol Venereol.

[12] Carlbring P, Andersson G, Cuijpers P, Riper H, Hedman-Lagerlöf E (2018). Internet-based vs. face-to-face cognitive behavior therapy for psychiatric and somatic disorders: an updated systematic review and meta-analysis. Cogn Behav Ther.

[13] Barak A, Hen L, Boniel-Nissim M, Shapira N (2008). A comprehensive review and a meta-analysis of the effectiveness of internet-based psychotherapeutic Interventions. J Technol Hum Serv.

[14] White V, Linardon J, Stone JE (2022). Online psychological interventions to reduce symptoms of depression, anxiety, and general distress in those with chronic health conditions: a systematic review and meta-analysis of randomized controlled trials. Psychol Med.

[15] Bundy C, Pinder B, Bucci S, Reeves D, Griffiths CEM, Tarrier N (2013). A novel, web-based, psychological intervention for people with psoriasis: the electronic Targeted Intervention for Psoriasis (eTIPs) study. Br J Dermatol.

[16] Moshe I, Terhorst Y, Philippi P (2021). Digital interventions for the treatment of depression: a meta-analytic review. Psychol Bull.

[17] Rothstein JD, Jennings L, Moorthy A (2016). Qualitative assessment of the feasibility, usability, and acceptability of a mobile client data app for community-based maternal, neonatal, and child care in rural Ghana. Int J Telemed Appl.

[18] (2016). Monitoring and evaluating digital health interventions: a practical guide to conducting research and assessment.

[19] Stuhlmann CFZ, Traxler J, Paucke V, da Silva Burger N, Sommer R (2025). Predictors and mechanisms of self-stigma in five chronic skin diseases: a systematic review. J Eur Acad Dermatol Venereol.

[20] Germain N, Augustin M, François C (2021). Stigma in visible skin diseases—a literature review and development of a conceptual model. J Eur Acad Dermatol Venereol.

[21] Gilbert P (2014). The origins and nature of compassion focused therapy. Br J Clin Psychol.

[22] Hudson MP, Thompson AR, Emerson LM (2020). Compassion-focused self-help for psychological distress associated with skin conditions: a randomized feasibility trial. Psychol Health.

[23] Ruwaard J, Lange A, Schrieken B, Emmelkamp P (2011). Efficacy and effectiveness of online cognitive behavioral treatment: a decade of interapy research. Stud Health Technol Inform.

[24] Bessell A, Brough V, Clarke A, Harcourt D, Moss TP, Rumsey N (2012). Evaluation of the effectiveness of Face IT, a computer-based psychosocial intervention for disfigurement-related distress. Psychol Health Med.

[25] Papadopoulos L, Bor R, Legg C (1999). Coping with the disfiguring effects of vitiligo: a preliminary investigation into the effects of cognitive-behavioural therapy. Br J Med Psychol.

[26] Braun TD, Olson K, Panza E (2022). Internalized weight stigma in women with class III obesity: a randomized controlled trial of a virtual lifestyle modification intervention followed by a mindful self-compassion intervention. Obes Sci Pract.

[27] Hopkins CM (2022). Reduction of internalized weight bias via mindful self-compassion: theoretical framework and results from a randomized controlled trial [Dissertation].

[28] Forbes YN, Moffitt RL, Van Bokkel M, Donovan CL (2020). Unburdening the weight of stigma: findings from a compassion-focused group program for women with overweight and obesity. J Cogn Psychother.

[29] Palmeira L, Cunha M, Pinto-Gouveia J (2019). Processes of change in quality of life, weight self-stigma, body mass index and emotional eating after an acceptance-, mindfulness- and compassion-based group intervention (Kg-Free) for women with overweight and obesity. J Health Psychol.

[30] Muftin Z, Gilbert P, Thompson AR (2022). A randomized controlled feasibility trial of online compassion-focused self-help for psoriasis. Br J Dermatol.

[31] Ewert C, Vater A, Schröder-Abé M (2021). Self-Compassion and coping: a meta-analysis. Mindfulness.

[32] Norman A, Veale J, Williamson H (2022). Assessing the usability and acceptability of Face IT@home: an online CBT intervention for people with visible differences. tCBT.

[33] Adkins KV, Overton PG, Thompson AR (2022). A brief online writing intervention improves positive body image in adults living with dermatological conditions. Front Med (Lausanne).

[34] Iliffe LL, Thompson AR (2019). Investigating the beneficial experiences of online peer support for those affected by alopecia: an interpretative phenomenological analysis using online interviews. Br J Dermatol.

[35] Krasuska M, Millings A, Lavda AC, Thompson AR (2018). Compassion-focused self-help for skin conditions in individuals with insecure attachment: a pilot evaluation of acceptability and potential effectiveness. Br J Dermatol.

[36] Kroenke K, Spitzer RL, Williams JBW, Löwe B (2009). An ultra-brief screening scale for anxiety and depression: the PHQ-4. Psychosomatics.

[37] Löwe B, Wahl I, Rose M (2010). A 4-item measure of depression and anxiety: validation and standardization of the Patient Health Questionnaire-4 (PHQ-4) in the general population. J Affect Disord.

[38] Pauley G, McPherson S (2010). The experience and meaning of compassion and self-compassion for individuals with depression or anxiety. Psychol Psychother.

[39] Lara MA, Patiño P, Tiburcio M, Navarrete L (2022). Satisfaction and acceptability ratings of a web-based self-help intervention for depression: retrospective cross-sectional study from a resource-limited country. JMIR Form Res.

[40] Boß L, Lehr D, Reis D (2016). Reliability and validity of assessing user satisfaction with web-based health interventions. J Med Internet Res.

[41] Kuijpers W, Groen WG, Loos R (2015). An interactive portal to empower cancer survivors: a qualitative study on user expectations. Support Care Cancer.

[42] Hurmuz MZM, Jansen-Kosterink SM, van Velsen L (2023). How to prevent the drop-out: understanding why adults participate in summative eHealth evaluations. J Healthc Inform Res.

[43] Han A, Kim TH (2023). Effects of self-compassion interventions on reducing depressive symptoms, anxiety, and stress: a meta-analysis. Mindfulness.

[44] Pedersen DH, Mansourvar M, Sortsø C, Schmidt T (2019). Predicting dropouts from an electronic health platform for lifestyle interventions: analysis of methods and predictors. J Med Internet Res.

[45] Melville KM, Casey LM, Kavanagh DJ (2010). Dropout from Internet-based treatment for psychological disorders. Br J Clin Psychol.

[46] Christensen H, Griffiths KM, Farrer L (2009). Adherence in internet interventions for anxiety and depression. J Med Internet Res.

[47] Jakob R, Harperink S, Rudolf AM (2022). Factors influencing adherence to mHealth apps for prevention or management of noncommunicable diseases: systematic review. J Med Internet Res.

[48] Van der Mispel C, Poppe L, Crombez G, Verloigne M, De Bourdeaudhuij I (2017). A self-regulation–based eHealth intervention to promote a healthy lifestyle: investigating user and website characteristics related to attrition. J Med Internet Res.

